# The Functional Significance of Synaptotagmin Diversity in Neuroendocrine Secretion

**DOI:** 10.3389/fendo.2013.00124

**Published:** 2013-09-18

**Authors:** Paanteha K. Moghadam, Meyer B. Jackson

**Affiliations:** ^1^Department of Neuroscience, University of Wisconsin, Madison, WI, USA

**Keywords:** exocytosis, neuropeptides, dense-core vesicle, norepinephrine, insulin, calcium, fusion pores, kiss-and-run

## Abstract

Synaptotagmins (syts) are abundant, evolutionarily conserved integral membrane proteins that play essential roles in regulated exocytosis in nervous and endocrine systems. There are at least 17 syt isoforms in mammals, all with tandem C-terminal C2 domains with highly variable capacities for Ca^2+^ binding. Many syts play roles in neurotransmitter release or hormone secretion or both, and a growing body of work supports a role for some syts as Ca^2+^ sensors of exocytosis. Work in many types of endocrine cells has documented the presence of a number of syt isoforms on dense-core vesicles containing various hormones. Syts can influence the kinetics of exocytotic fusion pores and the choice of release mode between kiss-and-run and full-fusion. Vesicles harboring different syt isoforms can preferentially undergo distinct modes of exocytosis with different forms of stimulation. The diverse properties of syt isoforms enable these proteins to shape Ca^2+^ sensing in endocrine cells, thus contributing to the regulation of hormone release and the organization of complex endocrine functions.

## Introduction

Nature employs the same basic molecular machinery for the release of both hormones and neurotransmitters (1–4). Several protein families function broadly in regulated exocytosis, including SNAREs, synaptotagmins (syts), and complexins (5–7). The rich molecular diversity within these families provides a platform for variations in the release process, enabling different cell types to tune and tailor release by blending the different molecular variants of the fusion apparatus. In this way endocrine cells can optimize secretory responses elicited by widely varying signals that are unique to each system. The rates of hormone release from different cell types vary by over two orders of magnitude (8). Endocrine cells secrete an extraordinary variety of hormones by exocytosis from dense-core vesicles (DCVs), and DCVs usually co-package collections of molecules ranging in size from small catecholamines to large peptides (9, 10). The nature of the stimulus can determine which packaged molecules will be released (11, 12), and a single DCV can release both catecholamines and neuropeptides simultaneously (13). Cells also can sort different hormones to different DCVs (14, 15). The diverse forms of storage and release raise questions as to how the exocytotic machinery can be called upon to modulate release rates and enable different types of Ca^2+^ signals to trigger the release of different substances from the same cell or even the same vesicle. One can hope to gain a better understanding of these problems by studying functional variations within the diverse families of exocytotic proteins.

Exocytosis proceeds through a sequence of distinct steps. Release can start once a fusion pore has formed to create a continuous fluid pathway from the vesicle interior to the extracellular space. The fusion pore is initially very small and can allow only small molecules such as norepinephrine to pass, but after it expands larger molecules such as chromogranins, insulin, and glucagon can escape. It is well established that DCVs of endocrine cells undergo two modes of exocytosis, kiss-and-run, and full-fusion (16–19). In kiss-and-run the pore opens transiently, and closes so that vesicles maintain their integrity as they retreat from the plasma membrane. The fusion pore formed during kiss-and-run can act as a filter to expel small molecules and retain larger molecules. The small molecules can be rapidly restored by vesicular transporters so DCVs can recycle. By contrast, in full-fusion the pore expands and the vesicle membrane collapses into the plasma membrane. After full-fusion a DCV is lost; replacing these DCVs requires the entire production sequence beginning with peptide hormone translation in the endoplasmic reticulum, DCV processing in the trans-Golgi network, and maturation (20). The choice between full-fusion and kiss-and-run thus plays a decisive role in determining the fate of the DCV as well as in selecting what molecules are released.

Ca^2+^ triggers exocytosis by binding to syt (21–23), but Ca^2+^ can also influence the kinetics of fusion pores in a variety of ways (24–26), thus raising the possibility that syt isoforms play roles not only in triggering exocytosis, but also in regulating release in subtle ways. Syts regulate the stability of fusion pores (26–30), and fusion pore stability in turn is intimately related to release mode, with the relative frequency of kiss-and-run versus full-fusion described quantitatively in terms of the kinetic rates of fusion pore closure and dilation (26). There are many examples of syts influencing the choice between kiss-and-run and full-fusion (26, 29, 31–33). Syts are conserved proteins with well established functions in membrane trafficking and exocytosis. They contain two C2 domains, and in syt 1, the first isoform to be characterized, each C2 domain binds two or three Ca^2+^ ions through interactions with key aspartate side chains (34). Syts interact with lipid membranes containing specific phospholipids including phosphatidylserine and phosphatidylinositol-4,5-biphosphate, as well as SNARE proteins. These interactions are regulated by Ca^2+^, but it remains unclear how binding to these targets enables syts to serve as Ca^2+^ sensors in exocytosis (21, 22, 30).

The mouse and human genomes encode 17 syt isoforms (35), and the functional significance of syt diversity is a subject of considerable interest (22, 36, 37). Syts vary widely in their Ca^2+^-dependent lipid binding, with syts 1, 2, and 3 binding rapidly, syts 5, 6, 9, and 10 binding at an intermediate rate, and syt 7 binding slowly (38). The isoforms also vary in their activity in Ca^2+^-stimulated liposome fusion: Ca^2+^ concentrations that trigger responses range widely between the isoforms, and many syts completely fail to confer Ca^2+^ sensitivity on SNARE-mediated liposome fusion *in vitro* (32, 39, 40). Variations in Ca^2+^ sensitivity and fusion pore regulation make these proteins ideal candidates for modifying the release apparatus and tuning responses as Ca^2+^ concentrations rise and fall in distinct spatiotemporal patterns. Here, we survey relevant work on syt functions in hormone release, and where possible draw parallels between syt isoform properties, Ca^2+^ signals, and forms of secretion.

## Syt Function in Endocrine Cells

Synaptotagmins appear broadly throughout the endocrine system, with essentially every cell type examined expressing multiple isoforms (Table 1). Expression varies between cell types and not all reports agree. No effort was made here to distinguish between isoforms untested versus undetected, and the number of isoforms found in endocrine cells will grow as reagents are developed and improved, and as proteomics methods advance. A large body of work supports the expression of syts 1, 4, 7, and 9 in many endocrine systems, and it is remarkable that these four molecules appear in so many different cell types. Syts 1, 4, and 7 are ancient, conserved proteins distributed widely through metazoan genomes (35). These isoforms presumably perform fundamental biological functions, and evidence is accumulating for their roles in a wide range of endocrine and non-endocrine systems.

**Table 1:** **Syt isoform expression in various endocrine systems**.

Cells	Syt isoforms	Reference
PC12 cells	1, 4, 7, 9	Tucker et al. (55)
	1, 9	Lynch and Martin (44), Fukuda et al. (54)
	3	Mizuta et al. (82)
	3, 5, 6, 10	Saegusa et al. (83)
	8	Monterrat et al. (68)
Chromaffin cells	1, 4, 7, 9	Matsuoka et al. (84)
	1, 7	Schonn et al. (47)
	1	Voets et al. (46)
Hypothalamus	1–4	Xi et al. (71)
Anterior pituitary	1, 3, 4	Xi et al. (71)
LβT2	1, 4	Hu et al. (85)
AtT20	3	Mizuta et al. (82)
	4	Eaton et al. (65)
GH3	3	Mizuta et al. (82)
Posterior pituitary	1, 4	Zhang et al. (63)
Intermediate pituitary (melanotrophs)	1, 3, 4, 7, 9	Kreft et al. (86)
Pancreatic islets	3, 4, 7	Gao et al. (87)
	3	Mizuta et al. (88)
	5, 9	Iezzi et al. (89)
	7	Gustavsson et al. (51)
Pancreatic β-cells	3	Brown et al. (90)
	4	Gut et al. (67)
	7	Gustavsson et al. (50)
β-Cell lines^a^	1–4, 7, 8	Gao et al. (87)
	1, 2	Lang et al. (91)
	3	Gut et al. (67), Mizuta et al. (82), Mizuta et al. (88)
	4, 7, 11, 13	Andersson et al. (69)
	5, 9	Iezzi et al. (89)
	8	Monterrat et al. (68)
Pancreatic α cells	7	Gustavsson et al. (51)

In most cases the localization and expression was based on immunocytochemistry (see text).

^a^ β-Cell lines include RINm5F, INS1, MN6, HT-T15, TC6-F7.

### Syt 1

Syt 1 is the most widely distributed syt isoform in nervous and endocrine systems. This low-affinity Ca^2+^ sensor (32, 39, 41, 42) generally triggers rapid exocytosis. The very tight temporal coupling between Ca^2+^ entry and fusion, within milliseconds, has prompted investigators to use the term “synchronous” to describe this form of release, particularly in the context of synaptic transmission. An early syt 1 knock-down study suggested that PC12 cells can secrete without syt 1 (43), but subsequent work showed that this was due to redundancy with another syt isoform of PC12 cells, syt 9 (33, 44). Overexpression of wild type syt 1 in PC12 cells left the overall time course of secretion unchanged, but overexpression of either wild type syt 1 or a number of syt 1 mutants altered fusion pore kinetics (26–28, 30, 45). Overexpressing syt 1 in PC12 cells also produced more kiss-and-run events than syt 7 and 9 (32).

Deletion of the syt 1 gene in mouse selectively abolished the initial rapid phase of exocytosis in chromaffin cells (33, 46, 47), but had no deleterious effects on slower Ca^2+^-triggered release. For a given concentration of Ca^2+^, exocytosis was much slower in chromaffin cells lacking syt 1 than in control cells (46). Mutation of a residue that reduces Ca^2+^ binding slowed exocytosis in chromaffin cells (48). In PC12 cells syt 1 sorted preferentially to smaller DCVs (32), raising the interesting possibility that hormones packaged in smaller vesicles will be released more rapidly than hormones packaged in larger vesicles.

### Syt 7

In contrast to syt 1, syt 7 acts as a high affinity Ca^2+^ sensor (32, 39, 49). Syt 7 is more abundant on larger DCVs in PC12 cells (32). Syt 7 overexpression in PC12 cells prolonged fusion pore lifetimes more than syt 1 overexpression (30), and favored full-fusion (32). Syt 7 knock-down in zebra fish reduced delayed synaptic release, indicating it is a slow Ca^2+^ sensor (49). Syt 7 deletion in chromaffin cells reduced Ca^2+^-triggered release by 50%, also selectively impairing the slow phase of exocytosis, and deletion of both syt 1 and 7 nearly abolished Ca^2+^-triggered exocytosis. Thus, in chromaffin cells syt 1 and 7 are the primary Ca^2+^ sensors for the fast and slow kinetic phases of exocytosis, respectively (47). Syt 7 gene ablation also reduced Ca^2+^-triggered exocytosis of insulin secretion from pancreatic β-cells and of glucagon secretion from pancreatic α-cells (50, 51). It is intriguing that syt 7 also functions in insulin responsive cells (fat and muscle), promoting glucose uptake through Ca^2+^-stimulated translocation of type-4 glucose transporter to the plasma membrane (52). In syt 7 knock-out α cells, ω-conotoxin further inhibited glucagon secretion to baseline levels, revealing the presence of an N-type Ca^2+^ channel-dependent component of residual glucagon secretion triggered by another protein (51).

### Syt 9

Syt 9 is closely related to syt 1 but exhibits intermediate Ca^2+^ sensitivity in fusion assays (32, 39). This protein has also been referred to as syt 5 (35, 53); here syt 9 refers to a 386 amino acid isoform. It is abundant on DCVs of PC12 cells (32, 54, 55), and overexpressing it produces fusion pore lifetimes intermediate between those seen with syt 1 and 7 (30). Down-regulating syt 9 alone produced a small, insignificant reduction of fusion rate in PC12 cells, but as noted above, because of the redundancy of syt 1 and 9 as Ca^2+^ sensors, both must be down-regulated to reduce secretion (33, 44). Silencing of syt 9 strongly inhibited insulin release from islet β-cells and INS-1E cells (56). However, mice with a pancreas-specific knock-out of syt 9 had normal glucose homeostasis and showed no changes in other insulin-dependent functions (57).

### Ca^2+^ non-binders

Slightly more than half of the mammalian syts have non-acidic amino acids at some of the positions engaged in Ca^2+^ binding (35), and these isoforms fail to act as Ca^2+^ sensors in liposome fusion assays (40). The best characterized of these, syt 4, harbors an evolutionarily conserved serine-for-aspartate substitution at a Ca^2+^ ligand in the C2A domain (58). Syt 4 is widely expressed in endocrine cells and its expression rises and falls depending on electrical activity (59) and reproductive state (60). Syt 4 negatively regulates both release and Ca^2+^-dependent liposome fusion (28, 32, 40) and does not bind Ca^2+^ (61). Syt 4 overexpression reduced exocytosis in PC12 cells (28, 32, 62). Although syt 4 overexpression in PC12 cells shortened the lifetimes of fusion pores capable of dilating to full-fusion (28), another form of release was enhanced in which very small fusion pores could open exclusively as kiss-and-run events with exceptionally long lifetimes. Many but not all of the effects of syt 4 overexpression were mimicked by syt 1 harboring the serine-for-alanine Ca^2+^ ligand replacement seen in syt 4 (29). Syt 4 overexpression also favored kiss-and-run in MIN6 β-cells (31). Ablation of the syt 4 gene altered exocytosis and fusion pore properties in posterior pituitary nerve terminals. These results suggested that syt 4 reduced exocytosis in response to modest Ca^2+^ rises but enhanced exocytosis in response to large Ca^2+^ rises (63). Syt 4 also contributes to the maturation of DCVs (64, 65), and altering syt 4 levels changes DCV size (62, 63, 66).

The Ca^2+^ non-binding isoforms syt 4, 8, and 13 have been detected in insulin-secreting cells (67–69). Silencing syt 4 and 13 reduced glucose stimulated insulin secretion in INS1 cells (69). Glucose stimulated expression of the syt 8 gene in human islets and syt 8 knock-down impaired both basal and evoked insulin release (70). Since syt 8 fails to stimulate liposome fusion in a Ca^2+^-dependent manner (40) the precise role of syt 8 in insulin secretion remains unclear. One intriguing possibility is that syt 8 increases the relative proportion of full-fusion events so that fusion pores can grow large enough to allow insulin to escape. It is likely that the Ca^2+^ non-binders interact with other components of the release machinery in ways that remain to be elucidated. These interactions could allow syts to regulate release in ways that cannot as yet be explained in terms of their biochemical properties.

Reports vary regarding the expression of other syt isoforms in endocrine cells (Table 1) and little is known about their localization and functions in hormone release. Syt 2 has been reported in the hypothalamus (71) but is absent from endocrine cells, and its primary function is likely to be as a synaptic Ca^2+^ sensor (72, 73).

## Specificity of Ca^2+^ Signaling

The differences in performance of syts as Ca^2+^ sensors gives a special significance to the spatiotemporal character of Ca^2+^ signals in different cell types and with different forms of stimuli. Ca^2+^ can enter cells through a variety of routes so that the dynamics and spatial extent of changes in cytosolic Ca^2+^ can vary enormously. This creates a scenario in which differences in Ca^2+^ sensing properties can have a major impact on responses (74). Intracellular Ca^2+^ rises and falls as Ca^2+^ enters through Ca^2+^ channels, diffuses away from these sources into the cytoplasm, binds Ca^2+^ binding proteins, and is sequestered into stores or pumped out of the cytoplasm (75–77). The opening of one voltage gated Ca^2+^ channel allows approximately 10^3^ ions to enter per msec. This localized flux sets up a steep gradient to create a domain of high local Ca^2+^ concentration. In the immediate vicinity of the channel mouth (within tens of nanometer) Ca^2+^ can rise to >100 μM, which is substantially higher than the average bulk cytoplasmic level, even under conditions of intense stimulation (75, 78). These Ca^2+^ domains around a Ca^2+^ channel can form and collapse rapidly (within a few ms) so that the activation of a Ca^2+^ sensor will depend critically on the kinetics of Ca^2+^ association and dissociation. A low-affinity sensor can detect this high local Ca^2+^ as long as it binds with rapid kinetics. A high affinity Ca^2+^ sensor may fail to respond if it binds too slowly. Syt 1 has properties suited for responding to transient domains with high Ca^2+^. Syt 7 has properties suited for responding to modest but prolonged rises in bulk Ca^2+^ concentration (38).

**Figure 1:**
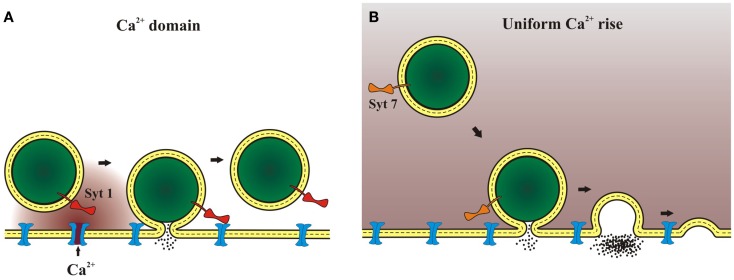
**Ca^2+^ domains and release mechanisms**. **(A)** Weak stimulation opens Ca^2+^ channels less frequently. One isolated channel can open and as Ca^2+^ flows a domain of high Ca^2+^ concentration will form around the channel mouth. This will lead to a highly localized Ca^2+^ signal that will persist for milliseconds. A Ca^2+^ sensor such as syt 1, with its rapid kinetics and low affinity, can be activated by a Ca^2+^ signal of this form. **(B)** Strong stimulation opens many Ca^2+^ channels to raise bulk Ca^2+^. Bulk Ca^2+^ can also rise as a result of Ca^2+^ release from internal stores. After the stimulus ends the Ca^2+^ gradients around individual vesicles will collapse as Ca^2+^ diffuses through the cytoplasm away from the membrane. This will lead to a more uniform, moderate concentration that can persist for seconds. A Ca^2+^ sensor such as syt 7, with its slow kinetics and high affinity, can be activated by a Ca^2+^ signal of this form.

The idea of localized Ca^2+^ signals leads to two types of Ca^2+^ concentration profile illustrated in Figure 1. When an endocrine cell fires at a moderate rate, Ca^2+^ domains form and collapse around a few open Ca^2+^ channels as shown in Figure 1A. The resulting brief period of high Ca^2+^ concentration will only affect vesicles near the open channels, and will preferentially activate syt 1 over slower isoforms. Basal firing rates corresponding to the resting state of an animal (feed and breed) have been shown to trigger norepinephrine release from chromaffin cells without triggering chromogranin release (11). This implicates exocytosis by kiss-and-run, which is preferentially triggered by syt 1 (32). By contrast, with vigorous electrical activity during stress (fight and flight) the bulk Ca^2+^ concentration will rise to moderate levels (well under 50 μM) for longer times on the order of seconds (Figure 1B). These signals will activate slow, low-affinity Ca^2+^ sensors such as syt 7. This form of [Ca^2+^] signal has been shown to trigger full-fusion (79), with release of both norepinephrine and chromogranin (11). This can be explained by the tendency of syt 7 to trigger full-fusion preferentially (32). Different spatiotemporal patterns of cytosolic Ca^2+^ can thus target different syt isoforms to modulate fusion kinetics. Furthermore, because syt isoforms favor different modes of exocytosis, by selectively promoting kiss-and-run or full-fusion, Ca^2+^ signals that activate different syts will determine the relative release of small and large molecules. The sorting of syts to different sized vesicles may also serve as a mechanism for allowing different Ca^2+^ signals to select different substances for release if content is also found to vary with vesicle size (32).

## Conclusion

Studies of how different syt isoforms influence the kinetics of exocytosis are starting to resolve functionally relevant distinctions in the mechanisms by which Ca^2+^ regulates exocytosis. More studies like these will expand our understanding of the diversity of endocrine release mechanisms, but rigorous assessments of syt isoform function in cells face a number of challenges. (1) It is important to address the expression of multiple isoforms, either by ablating endogenous proteins or varying each isoform individually with careful assessment of protein levels. Overexpression of proteins can result in high protein concentrations, possibly leading to mis-targeting and artificial functions. (2) Experiments need to address the different Ca^2+^ sensitivities of syt isoforms. This requires measurement and control of Ca^2+^ concentration. (3) Biophysical techniques for measuring exocytosis are very sensitive but often measure surrogates of release and detect events on different time scales. Amperometry measures release and can detect rapid processes but its greatest sensitivity is realized with biogenic amines, and is not nearly as powerful in the measurement of peptides. Furthermore, after content expulsion the closure of a fusion pore can no longer be detected, and the mode of release as kiss-and-run or full-fusion can no longer be ascertained. Capacitance measures membrane area, and single-vesicle capacitance steps provide strong evidence for kiss-and-run. Total internal reflectance microscopy of fluorescent tracers follows the fate of the vesicle content or membrane (80). Like capacitance recording, this method can reveal kiss-and-run but its time resolution is poor. Furthermore, the fate of fluorescent cargo can vary with the design of the fusion construct, raising concerns about experimental artifacts (81). Amperometry, capacitance, and total internal reflectance microscopy are very powerful techniques but they do not always agree and these differences can complicate interpretations and comparisons (62).

Given the functional versatility within the syt protein family, the studies to date probably have only scratched the surface in addressing the roles of syt isoforms in the complex and varied forms of hormone release. By selecting among the different syt isoforms and regulating their expression, and by sorting to different vesicles containing different hormones, cells can regulate the response to diverse forms of Ca^2+^ signals. The syt isoforms present on a vesicle will determine what form of Ca^2+^ signal will trigger fusion, how different Ca^2+^ signals direct the choice between kiss-and-run and full-fusion, and what proportion of small and large molecules are released.

## Conflict of Interest Statement

The authors declare that the research was conducted in the absence of any commercial or financial relationships that could be construed as a potential conflict of interest.
